# The implementation of person-centred plans in the community-care sector: a qualitative study of organizations in Ontario, Canada

**DOI:** 10.1186/s12913-024-11089-7

**Published:** 2024-05-29

**Authors:** Samina Idrees, Gillian Young, Brian Dunne, Donnie Antony, Leslie Meredith, Maria Mathews

**Affiliations:** 1 Department of Family Medicine, Schulich School of Medicine & Dentistry, Western University, 1151 Richmond St, London, ON N6A 5C1 Canada; 2 PHSS - Medical & Complex Care in Community, 620 Colborne St, London, ON N6B 3R9 Canada

**Keywords:** Person-centred planning, Community-based care, Integrated care, Social services, Health services, Disability, Organizational culture, Qualitative study

## Abstract

**Background:**

Person-centred planning refers to a model of care in which programs and services are developed in collaboration with persons receiving care (i.e., persons-supported) and tailored to their unique needs and goals. In recent decades, governments around the world have enacted policies requiring community-care agencies to adopt an individualized or person-centred approach to service delivery. Although regional mandates provide a framework for directing care, it is unclear how this guidance is implemented in practice given the diversity and range of organizations within the sector. This study aims to address a gap in the literature by describing how person-centred care plans are implemented in community-care organizations.

**Methods:**

We conducted semi-structured interviews with administrators from community-care organizations in Ontario, Canada. We asked participants about their organization’s approach to developing and updating person-centred care plans, including relevant supports and barriers. We analyzed the data thematically using a pragmatic, qualitative, descriptive approach.

**Results:**

We interviewed administrators from 12 community-care organizations. We identified three overarching categories or processes related to organizational characteristics and person-centred planning: (1) organizational context, (2) organizational culture, and (3) the design and delivery of person-centred care plans. The context of care and the types of services offered by the organization were directly informed by the needs and characteristics of the population served. The culture of the organization (e.g., their values, attitudes and beliefs surrounding persons-supported) was a key influence in the development and implementation of person-centred care plans. Participants described the person-centred planning process as being iterative and collaborative, involving initial and continued consultations with persons-supported and their close family and friends, while also citing implementation challenges in cases where persons had difficulty communicating, and in cases where they preferred not to have a formal plan in place.

**Conclusions:**

The person-centred planning process is largely informed by organizational context and culture. There are ongoing challenges in the implementation of person-centred care plans, highlighting a gap between policy and practice and suggesting a need for comprehensive guidance and enhanced adaptability in current regulations. Policymakers, administrators, and service providers can leverage these insights to refine policies, advocating for inclusive, flexible approaches that better align with diverse community needs.

**Supplementary Information:**

The online version contains supplementary material available at 10.1186/s12913-024-11089-7.

## Background

The community-care sector facilitates the coordination and administration of in-home and community-based health and social services. Community-care services include supports for independent living, residential services, complex medical care, and community-participation services to support personal and professional goals (e.g., education, employment, and recreation-based supports) [1]. There is substantial heterogeneity in the clinical and demographic characteristics of the community-care population, including individuals with physical and developmental disabilities, and complex medical needs [2]. We refer to the individuals served by these organizations as ‘persons-supported’ in line with person-first language conventions [3, 4].

In recent decades, governments across the world have enacted policies requiring community-care agencies to adopt an individualized or person-centred approach to service delivery [5–8]. Person-centred care encompasses a broad framework designed to direct care delivery, as opposed to a singular standardized process. In the context of community-care, person-centred planning refers to a model of care provision in which programs and services are developed in collaboration with persons-supported and tailored to their unique needs and desired outcomes [9, 10].

In Ontario, Canada, community-care services are funded by the Ministry of Health (MOH) and the Ministry of Children, Community and Social Services (MCCSS). Service agreements between these ministries and individual agencies can be complex and contingent on different factors including compliance with a number of regulatory items and policies [7, 11]. MOH provides funding for health-based services including in-home physiotherapy, respiratory therapy, and personal support services, among several others. MOH funds Home and Community Care Support Services (HCCSS), a network of organizations responsible for coordinating the delivery of in-home and community-based care in the province. MCCSS funds social service agencies including those providing community participation and residential support for people with intellectual and developmental disabilities (IDDs).

Several tools and resources have been developed to aid organizations in providing person-centred care and organizations may differ in their use of these tools and their specific approach. Although regional mandates provide a framework for directing care delivery, it is unclear how this guidance is implemented in practice given the diversity and range of organizations within the sector. In addition, as noted by a recent scoping review, there is limited literature on the implementation process and impact of person-centred planning on individual outcomes [12]. Using a pragmatic, qualitative, descriptive approach [13], we outline how community-care organizations enact a person-centred approach to care and the factors that shape their enactment. By describing existing practices in the context of the community-care sector, we aim to provide insight on how to optimize care delivery to improve outcomes and inform current policy. This study is part of a larger, multi-methods project examining the implementation of person-centred care plans in the community-care sector. This project encompasses qualitative interviews with representatives from different community-care organizations, as well as staff and persons-supported at a partner community-care organization. This paper focuses on analyzing data from interviews with representatives from different community-care organizations.

## Methods

We conducted semi-structured interviews with administrators from community-care organizations in Southwestern Ontario (roughly the Ontario Health West Region) between October 2022 and January 2023. We included community-care organizations funded by MOH or MCCSS. We excluded organizations that did not provide services in Southwestern Ontario. We identified eligible organizations and participants by searching online databases, including community resource lists, as well as through consultation with members of the research team.

We used maximum variation sampling [14], to recruit participants from organizations with a wide range of characteristics including location (i.e., urban, rural), organization type (i.e., for-profit, not-for-profit), and types of services provided (e.g., residential, recreation, transportation, etc.) We contacted eligible organizations via email, providing them with study information and inviting them to participate. We recruited until the data reached saturation, defined as the point at which there was sufficient data to enable rigorous analysis [14, 15].

In each interview, we asked participants about their organization’s approach to developing and updating individual service agreements or person-centred care plans, and the supports and barriers (e.g., organizational, funding, staffing, etc.) that facilitate or hinder the implementation of these plans (Supplementary Material 1: Interview Guide). We also collected information on relevant participant and organizational characteristics, including participant gender, position, years of experience, organization location, type (i.e., for-profit, not-for-profit), services offered, years in operation, and client load. The interviews were approximately one hour in length and conducted virtually via Zoom (Zoom Video Communications Inc.) or by telephone. The interviews were audio-recorded and transcribed verbatim. Interviewer field notes were also used in data analysis.

We analyzed the data thematically [16]. The coding process followed a collaborative and multi-step approach. Initially, three members of the research team independently reviewed and coded a selection of transcripts to identify key ideas and patterns in the data, and form a preliminary coding template. We then met to consolidate individual coding efforts. We compared coding of each transcript, resolving conflicts through discussion and consensus. In coding subsequent transcripts and through a series of meetings, we worked together to finalize the codebook to reflect more analytic codes. We used the finalized template to code all interview transcripts in NVivo (QSR International), a software designed to facilitate qualitative data analysis. We refined the codebook on an as-needed basis by incorporating novel insights gleaned from the coding of additional transcripts, reflecting the iterative nature of the analysis.

We increased the robustness of our methodology by pre-testing interview questions, documenting interview and transcription protocols, using experienced interviewers, and confirming meaning with participants in interviews [14–16]. We kept detailed records of interviews, field notes, and drafts of the coding template. We made efforts to identify negative cases and provided rich descriptions and illustrative quotes [17]. We included individuals directly involved in the administration of community-care services on our research team. These individuals provided important context and feedback at each stage of the research process.

This study was approved by the research ethics board at Western University. We obtained informed consent from participants prior to the onset of interviews. We maintained confidentiality through secure storage of interview data (e.g., audio recordings), password-protection of sensitive documents, and the de-identification of transcripts.

### Positionality

The authors represent a multidisciplinary team of researchers, clinicians, and community-care leaders. The community-care leaders and clinicians on our team provided key practical expertise to inform the development of interview questions and the analysis of study findings.

## Results

We interviewed administrators across 12 community-care organizations in Southwestern Ontario. The sample included representatives from seven organizations that received funding from MCCSS, three organizations that received funding from MOH, and two organizations that received funding from both MCCSS and MOH (Table 1). Eleven organizations were not-for-profit, one was a for-profit agency. The organizations provided care in rural (*n =* 3), urban (*n =* 4), or both rural and urban populations (*n =* 5). Seven of the 12 participants were women, nine had been working with their organization for more than 11 years, and all had been working in the community-care sector for more than 12 years (Table 2).

**Table 1:** Characteristics of participating organizations (*N* = 12)

Primary funder, *n* (%)	
MCCSS	7 (58.3)
MOH	3 (25.0)
MOH and MCCSS	2 (16.7)
Organization model, n (%)	
Not-for-profit	11 (91.7)
For-profit	1 (8.3)
Population served, n (%)	
Individuals with IDD	8 (66.7)
Individuals with ABI	1 (8.3)
Individuals with IDD and/or ABI	1 (8.3)
Individuals with complex health needs	2 (16.7)
Location of service provision, n (%)	
Rural	3 (25.0)
Urban	4 (33.3)
Rural and urban	5 (41.7)

Abbreviations: MOH: Ministry of Health; MCCSS: Ministry of Children, Community and Social Services; IDD: intellectual or developmental disability; ABI: acquired brain injury

**Table 2:** Characteristics of study participants (*N* = 12)

Gender, n (%)	
Woman	7 (58.3)
Man	4 (33.3)
Missing	1 (8.3)
Current role, n (%)	
Executive Director	6 (50.0)
Associate Director	1 (8.3)
Director of Services	4 (33.3)
Residential Supervisor	1 (8.3)
Years in current role, mean (SD)	6.7 (5.1)
Missing, n (%)	1 (8.3)
Years with the organization, mean (SD)	15.0 (10.9)
Years working in community-care, mean (SD)	26.8 (10.2)
Missing, n (%)	3 (25.0)

We identified three key categories or processes relating to organizational characteristics and their impact on the design and delivery of person-centred care plans: (1) organizational context, (2) organizational culture, and (3) the development and implementation of person-centred care plans.

### Organizational context

Organizational context refers to the characteristics of persons-supported, and the nature of services provided. Organizational context accounts for the considerable heterogeneity across organizations in the community-care sector and their approach to person-centred care plans.

#### Populations served

The majority of organizations included in the study supported individuals with IDDs: *“all of the people have been identified as having a developmental disability. That’s part of the eligibility criteria for any funded developmental service in Ontario.”* [P10]. Participants described how eligibility was ascertained through the referral process: “*the DSO [Developmental Services Ontario] figures all of that out and then refers them to us*.” [P08]. These descriptions highlighted a common access point for publicly-funded adult developmental services in the province. Accordingly, these organizations were primarily funded by MCCSS. Other organizations focused on medically complex individuals including those with acquired brain injuries or those unable to access out-patient services due to physical disabilities: *“the typical reason for referral is going to be around a physical impairment… But, with this medically complex population, you’re often seeing comorbidities where there may be some cognitive impairment, early dementia.”* [P04]. In these organizations, eligibility and referral were usually coordinated by HCCSS. These insights highlighted the diverse characteristics of community-care populations, emphasizing the need to consider both physical and cognitive health challenges in care provision approaches.

#### Services offered

The characteristics of persons-supported informed the context of care and the type of services offered by the organization. The different dimensions of services offered within this sector include social and medical care, short and long-term care provision, in-home and community-care, and full and part-time care.

##### Nature of care: social vs. medical

Many organizations serving individuals with IDDs employed a holistic, psychosocial model of care, designed to support all areas of an individual’s life including supports for independent-living, and community-based education, employment, and recreation services to support personal and professional goals: *“we support people in their homes, so residential supports. We also support people in the community, to be a part of the community, participate in the community and also to work in the community.”* [P06]. These descriptions reflect a comprehensive approach to care, aiming to address needs within and beyond residential settings to promote active participation within the broader community. In contrast, some organizations followed a biomedical model of care, designed to support specific health needs: *“We provide all five therapies… physiotherapy, occupational therapy, speech, social work, and nutrition. In some locations we provide visiting nursing, at some locations shift nursing. We have some clinic-nursing… and we provide personal support and home-making services in a number of locations as well.”* [P04]. These organizations adopted a more clinically-focused approach to care. In either instance, the care model and the nature of services offered were largely determined by an organization’s mandate including which gaps they aimed to fill within the community. Many organizations described providing a mixture of social and medical care for individuals with complex needs. However, the implementation of care plans could be impacted by the lack of integration between social and medical care sectors, as some participants spoke to the importance of *“[integrating] all of the different healthcare sector services… [including] acute care and public health and home and community care and primary care, and mental health and addictions.”* [P04].

##### Duration of care: short-term vs. long-term

The duration of care also varied based on the needs of persons-supported. Organizations serving individuals with IDDs usually offered support across the lifespan: *“We support adults with developmental disabilities and we support them from 18 [years] up until the end of their life.”* [P06]. Some organizations provided temporary supports aimed at addressing specific health needs: *“For therapies – these are all short-term interventions and typically they’re very specific and focused on certain goals. And so, you may get a referral for physiotherapy that is authorized for three visits or five visits”* [P04], or crisis situations (e.g., homelessness): *“Our services are then brought in to help provide some level of support, guidance, stabilization resource, and once essentially sustainability and positive outcomes are achieved—then our services are immediately withdrawn.”* [P12]. One organization employed a model of care with two service streams, an initial rehabilitation stream that was intended to be short-term and an ongoing service stream for individuals requiring continuing support.

##### In-home vs. community-based care

Many organizations provided in-home care and community-based supports, where residential supports were designed to help individuals lead independent lives, and community-based supports encouraged participation in community activities to further inclusion and address personal and professional goals. One participant spoke about the range of services offered in the home and community:

*“There’s probably two big categories of [services we offer]: community support services—so that includes things like adult day programs, assisted living, meals on wheels, transportation, friendly visiting … and things like blood pressure clinics, exercise programs… and then on the other side we do home care services. In the home care basket, we provide personal support, and we also provide social work support.”* [P05].

Likewise, another participant spoke in further detail on the types of services that allow individuals to live independently within their homes, or in community-based residential settings (e.g., long-term care facilities):

*“We provide accommodation supports to about 100 people living in our community—which means that we will provide support to them in their own homes. So, anywhere from an hour a week to 24 hours a day. And that service can include things from personal care to home management to money management, cooking, cleaning, and being out and about in communities—so community participation. We also provide supports for about 50 people living in long-term care facilities and that is all community participation support. So, minus the last 2 and a half years because of the pandemic, what that means is that a person living in a long-term care facility with a developmental disability can have our support to get out and about for 2 or 3 hours a week, on average.”* [P10].

##### Full-time vs. part-time support

The person-supported’s needs also determined whether they would receive care within their homes and if they would be supported on a full-time (i.e., 24 h a day, 7 days a week) or part-time basis:

“*It really does range from that intensive 24- hour/7 day a week support, which we actually do provide that level of intense support in the family home, if that’s needed. And then, all the way through to just occasional advocacy support and phone check-in.”* [P01].

### Organizational Culture

Organizational culture was described as a key influence in the development and implementation of person-centred care plans. The culture of the organization includes their perceptions, attitudes and beliefs surrounding persons-supported; their model of care provision; as well as their willingness to evolve and adapt service provision to optimize care delivery.

#### Perceptions, attitudes, and beliefs regarding persons-supported

Participants described their organization’s view of persons-supported, with many organizations adopting an inclusionary framework where persons-supported were afforded the same rights and dignities as others in the community. This organizational philosophy was described as being deeply intertwined with an organization’s approach to personalizing programs and services:

*“…an organization needs to be able to listen to the people who are receiving the service… and support them, to learn more, figure out, articulate, whatever it is, the service or the supports that they need in order to get and move forward with their life.”* [P10].

The focus on the person-supported, their needs, likes, and dislikes, was echoed across organizations, with an emphasis on the impact of *“culture and trying to embed for each person who delivers service the importance of understanding the individual.”* [P05]. Participants also described their organization’s approach to allowing persons-supported to take risks, make mistakes, and live life on their own terms:

*“You have to go and venture out and take some [risks]… We try to exercise that philosophy - people with disabilities should have the same rights and responsibilities as other people in the community. Whether that’s birthing or education, getting a job, having a house they can be proud of, accessing community supports, whether that be [a] library or community centre, or service club, whatever that is.”* [P03].

#### Model of care provision

The model of care provision was heavily influenced by the organization’s values and philosophy. Several organizations employed a flexible model of care where supports were developed around the needs, preferences, and desired outcomes of the person-supported:

*“…if we don’t offer [the program they want], we certainly build it. Honestly, most of our programs were either created or built by someone coming to us [and] saying ‘I want to do this with my life,’ or …‘my son would like to do art.’”* [P02].

Although there were similarities in models across the different organizations, one participant noted that flexibility can be limited in the congregate care setting as staff must tend to the needs of a group as opposed to an individual:

*“Our typical plan of operation outside of the congregate setting is we design services around the needs of the person. We don’t ask them to fit into what we need, we build services for what they need. Within the congregate care setting, we have a specific set of rules and regulations for safety and well-being of the other people that are here.”* [P11].

#### Evolving service orientation

In organizations serving individuals with IDDs, many described shifting from program-based services to more individualized and community-based supports: *“The goal was always to get people involved in their community and build in some of those natural supports … [we] are looking to support people in their own communities based on their individual plans.”* [P07]. One participant described this model as a person-directed approach as opposed to person-centred, citing the limitations of program-based services in meeting individual needs:

*“[Persons-supported] couldn’t [do] what they wanted because they were part of a bigger group. We would listen to the bigger group, but if one person didn’t want to go bowling … we couldn’t support them because everybody had to go bowling.”* [P06].

The focus on individualized support could potentially lead to increased inclusion for persons-supported in their communities:

*“… people go to Tim Horton’s, and if they go every day at 9 they probably, eventually will meet other people that go at 9 o’clock and maybe strike up a conversation and get to know somebody and join a table … and meet people in the community.”* [P02].

By creating routines centred on individual preferences, the person-supported becomes a part of a community with shared interests and values.

### Person-centred care plans

Community-care organizations enacted a person-centred approach by creating person-centred care plans for each person-supported. Although all participants said their organization provided person-centred services, there was considerable variation in the specific processes for developing, implementing, and updating care plans.

#### Developing a person-centred care plan

The development of a care plan includes assessment, consultation, and prioritization. The initial development of the care plan usually involved an assessment of an individual’s needs and goals. Participants described agency-specific assessment processes that often incorporated information from service referrals: “*In addition to the material we get from the DSO [Disability Services Ontario] we facilitate the delivery of an intake package specifically for our services. And that intake package helps to further understand the nature and needs of an individual.”* [P12]. Agency-specific assessment processes differed by the nature of services provided and the characteristics of the population. However, most organizations included assessments of *“not only physical functioning capabilities, but also cognitive.”* [P01]. Assessment also included an appraisal of the suitability of the organization’s services. In instances where persons-supported were seeking residential placements or independent-living support, organizations assessed their ability to carry out the activities of daily living:

*“[Our internal assessment] is an overview of all areas of their life. From, ‘do they need assistance with baking, cooking, groceries, cleaning, laundry? Is there going to be day program opportunities included in that residential request for placement? What the medical needs are?’”* [P02].

In contrast, the person-supported’s community-based activities were primarily informed by their interests and desired outcomes: *“We talk about what kinds of goals they want to work on. What kind of outcomes we’re looking for…”* [P06].

The development of the care plan also included a consultation phase, involving conversations with the person-supported, their family members, and potentially external care providers: *“We would use the application information, we’d use the supports intensity scale, but we’d also spend time with the person and their connections, their family and friends, in their home to figure out what are the kinds of things that this person needs assistance with.”* [P10]. Participants described the person-supported’s view as taking precedence in these meetings: *“We definitely include the family or [alternate] decision-maker in that plan, but the person-supported ultimately has the final stamp of approval.”* [P08]. Many participants also acknowledged the difficulty of identifying and incorporating the person-supported’s view in cases where opinions clash and the person-supported has difficulty communicating and/or is non-verbal: *“Some of the people we support are very good at expressing what they want. Some people are not. Some of our staff are really strong in expressing what they support. …And some of the family members are very strong. So you have to be very careful that the [person-supported] is not being lost in the middle of it.”* [P06].

Participants also noted that some persons-supported preferred not to have a care plan:

*“Some of the people say ‘I hate [the plans] I don’t want to do them’…. we look at it in a different way then. We’ll use graphic art, we’ll use video, we’ll think outside the box to get them to somehow—because at the end of the day when we’re audited by MCCSS every [person-supported] either has to have [a plan]… or there has to be [an approval of] why it wasn’t completed.”* [P02].

Plan development may also include a prioritization process, particularly in cases where resources are limited. A person-supported’s goals could be prioritized using different schemas. One participant noted that *“the support coordinator takes the cue from the person-supported - … what they’ve identified as ‘have to have’ and ‘nice to have’. … because the ‘have to haves’ are prioritized.”* [P09]. Likewise, the person-supported’s preference could also be identified through *“[an] exercise, called ‘what’s important for and what’s important to*.’” [P06]. This model, based on a Helen Sanderson approach [18], was described as being helpful in highlighting what is important *to* the person-supported, as opposed to what others (i.e., friends, family, staff, etc.) feel is important *for* them.

Several organizations updated care plans throughout the year, to document progress towards goals, adapt to changing needs and plan for future goals: *“We revisit the plan periodically through the year. And if they say the goal is done, we may set another goal.”* [P06]. Organizations may also change plans to adapt to the person-supported’s changing health status or personal capacity.

#### Implementing a person-centred care plan

The implementation of care plans differed based on the nature of services provided by the organization. The delivery of health-based or personal support services often involved matching the length and intensity of care with the individual’s needs and capacity:

*“Sometimes that is a long time, sometimes it’s a short time, sometimes it’s an intervention that’s needed for a bit, and then the person is able to function.”* [P05].

In contrast, the delivery of community-based services involved matching activities and staff by interests: *“[if] a person-supported wants to go out and be involved in the music community, then we pull the staff pool in and match them up according to interest.”* [P06].

Broad personal goals were broken down into smaller, specific activities. For example, one participant described their organization’s plan in helping a person-supported achieve his professional goal of securing employment:

*“[The person-supported] said ‘Okay, I want a job.’ So for three weeks he was matched up with a facilitator. They came up with an action plan in terms of how to get a job, what kind of job he’s looking for, where he wants to go, where he wants to apply, how to conduct an interview. And after three weeks he got a job.”* [P09].

Organizations that provided residential services focused on developing independent-living skills. One participant described their organization’s plan to empowering persons-supported by allowing them to make their own financial decisions:

*“If one month they’re looking after their own finances, and they’ve overspent. Well, maybe we help them out with a grocery card or something and say ‘okay, next month how are you going to do this?’ [The person-supported may say], ‘well, maybe I’ll put so much money aside each week rather than doing a big grocery shop the first week and not having enough money left at the end of the month.’”* [P03].

The participant noted that *“a tremendous amount of learning [happens] when a person is allowed to [take] risks and make their own decisions.”* [P03].

Likewise, participants representing organizations that provided residential services described tailoring care to the persons-supported’s sleeping schedule and daily routine:

*“We develop a plan and tweak it as we go. With [the person-supported] coming to the home, what worked well was, we found that he wanted to sleep in, so we adjusted the [staff] time. We took a look at his [medication] times in the morning… and [changed] his [medication] times. We found that he wanted to sleep [until] later in the day, so he would get up at 10 o’clock, so then instead of having breakfast, lunch, and supper he would just have a bigger brunch. Just really tailoring the plan around the person-supported, and it’s worked out well.”* [P08].

These examples highlight how organizational context and culture influence how organizations operationalize person-centred care plans; the same individual may experience different approaches to care and engage in different activities depending on the organization they receive services from.

## Discussion

In this paper, we described key elements of the person-centred planning process across different community-care organizations in Southwestern Ontario. We also identified that the context and culture of an organization play a central role in informing the process by which services are personalized to an individual’s needs. These findings shed light on the diversity of factors that influence the implementation of person-centred care plans and the degree to which organizations are able to address medical and social needs in an integrated fashion. They also inform future evaluations of person and system-related outcomes of person-centred planning.

There are regulations around individualizing services delivered by community-care organizations, whereby care providers must allow persons-supported to participate in the development and evaluation of their care plans. HCCSS or MOH-funded services are largely focused on in-home rehabilitation or medical care. In contrast, MCCSS-funded organizations often focus on developing independent living skills or promoting community participation, thus highlighting the role of the funding agency in determining organizational context as well as the nature of services and personalization of care plans.

We also identified organizational culture as a key influence in the person-centred planning process. In previous reports, organizational culture, and specifically the way in which staff perceive and view persons-supported and their decision-making capabilities can impact the effective delivery of person-centred care [19]. Staff support, including their commitment to persons-supported and the person-centred process, has been regarded as one of the most powerful predictors of positive outcomes and goal attainment in the developmental services sector [20, 21]. Moreover, in order to be successful, commitment to this process should extend across all levels of the organization, be fully integrated into organizational service delivery, and be reflected in organizational philosophy, values and views of persons-supported [22–24].

MCCSS mandates that agencies serving individuals with IDDs develop an individual service plan (ISP) for each person-supported, one “that address[es] the person’s goals, preferences and needs.” [7]. We reference ISPs as person-centred care plans, as is in line with the view of participants in interviews. There are a series of checklists designed to measure compliance with these policies, and the process is iterative, with mandated annual reviews of care plans and active participation by the person-supported [25]. In our study, the agencies funded by MCCSS adhered to the general framework outlined by these regulations and informed service delivery accordingly. However, participants also described areas for improvement with respect to the implementation of these policies in practice. These policies, while well-intentioned, may imply a one-size-fits-all approach and appear more as an administrative exercise as opposed to a meaningful endeavor designed to optimize care. Participants spoke about individuals who preferred not to have an ISP, and how that in and of itself is a person-centred approach, respecting the person’s wishes. Additionally, we heard about how the goal-setting process may not be realistic as it can be perceived as unnatural to have goals at each point in one’s life. Moreover, participants noted challenges in implementing person-centred care in shared residential settings (e.g., group homes) or in cases where persons-supported had difficulty communicating.

Prior research indicates that individuals living in semi-independent settings fare better across several quality-of-life measures relative to individuals living in group homes, including decreased social dissatisfaction, increased community participation, increased participation in activities of daily living, and increased empowerment [26]. Furthermore, a recent study by İsvan et al. (2023) found that individuals living in the community (e.g., own home, family home, or foster home) exhibit greater autonomy in making everyday and life decisions, and greater satisfaction with their inclusion in the community [27]. These findings may be indicative of a reduced focus on person-centred care plan development and implementation in congregate care settings, where limited staff capacity can make it difficult to tend to the needs of everyone in the home. However, poor outcomes may also be explained by potentially more complex health challenges or more severe disability in persons-supported living in congregate care settings. The challenges described in our study are consistent with calls to improve the quality of care provided in residential group home settings [28, 29].

In line with our findings, previous literature also describes challenges in implementing person-centred planning for individuals who have difficulty communicating or are non-verbal [19, 30–32]. Communication has also been identified as a barrier to patient-centred care for adults with IDDs in healthcare settings [33, 34]. Other reports have identified a need for increased training and awareness of diverse communication styles (including careful observation of non-verbal cues) to aid staff in including persons-supported in the development of care plans [35–37]. Importantly, these methods take substantial time which is often limited, and compounded by staffing shortages that are widespread across the sector [38]. Similar barriers were identified in interviews with staff and persons-supported at a partner community-care agency within our larger project [39]; other papers from the project examine strategies used by the organization to overcome these barriers.

### Limitations

The findings from this study should be interpreted in the context of the following limitations. There is a risk for social desirability bias, whereby participants may feel pressure to present their care plan process in a more positive light due to societal norms and expectations [40]. Additionally, the experiences and views of community-care organizations may vary by region and organization type (i.e., for-profit vs. not-for-profit). In this study, we limited participation to agencies providing services in Southwestern Ontario and we were only able to interview one for-profit agency, despite concerted recruitment efforts. Consequently, we may not have fully captured how financial pressures, or different contextual and cultural components of an organization impact their implementation of care plans.

## Conclusions

The person-centred planning process in community-care organizations is largely informed by the characteristics of the population served and the nature of services offered (i.e., organizational context). This process usually involves initial and continued consultations with persons-supported to tailor plans to their specific needs and desired outcomes. There are ongoing challenges in the implementation of person-centred planning, including a need for increased adaptability and clarity in current regulations. In some areas, there may be benefit to incorporating nuance in the application of policies (e.g., in cases where a person-supported does not want to have a formal plan in place). In other areas, it may be helpful to have increased guidance on how to optimize care delivery to improve outcomes (e.g., in cases where a person-supported has difficulty communicating, or is residing in a group home). Policymakers, administrators, and service providers can leverage these insights to refine policies, advocating for inclusive, flexible approaches that better align with diverse community needs.

### Electronic supplementary material

Below is the link to the electronic supplementary material.

Supplementary Material 1

## Data Availability

The datasets generated and analyzed in the current study are not publicly available to maintain participant confidentiality, however access may be granted by the corresponding author upon reasonable request.

## Abbreviations

ABI: Acquired Brain Injury
DSO: Disability Services Ontario
HCCSS: Home and Community Care Support Services
IDDs: Intellectual and Developmental Disabilities
ISP: Individual Service Plan
MCCSS: Ministry of Children, Community and Social Services
MOH: Ministry of Health
