# Supplementary material for: The implementation of person-centred plans in the community-care sector: a qualitative study of organizations in Ontario, Canada
Source: BMC Health Serv Res. 2024 May 29;24:680. doi: 10.1186/s12913-024-11089-7 (PMC11137948; doi:10.1186/s12913-024-11089-7)
Supplement: Supplementary file 1 — Supplementary Material 1 [file 12913_2024_11089_MOESM1_ESM.pdf]

Supplementary Material 1: Interview Guide

General Introduction

Thank you for taking the time to speak with me today. We are conducting this study to understand how person-centred care plans or individual service plans are determined in the community-care sector. We are hoping you will tell us about your organization, and its approach to developing these plans.

This interview should take us approximately one hour to complete. The interview will be recorded and transcribed. You will not be identified in any report or presentation; your name will be replaced by a participant code and any identifying information will be removed.

Participation is voluntary. You may end the interview at any time or choose not to answer any specific question. Any questions before we begin?

Administrators of Community-Care Organizations Interview Guide

First, I would like to ask some general background questions.

1. Please tell me your job title and your role in the organization.
2. How long have you been in this position?
    a. How long have you been with this organization?
    b. How long have you been working in the community-care sector?
3. How would you describe your gender?

Next, I would like to ask some general background questions about your organization.

4. What term is used in your organization to describe the people you support?
    a. Patient, client, person supported.
5. Please tell me a bit about this organization.
    a. What types of services does this organization provide?
        i. Probe: residential care, transportation services, recreation, health and wellness.
    b. How long has this organization been operating?
    c. How would you describe the general population and demographics of the [patients/clients/persons-supported] you serve?
        i. Probe: age, sex, gender, underlying conditions, residences, and community size.
    d. How many [patients/clients/persons-supported] does this organization serve?
        i. Day-to-day?
        ii. In a year?
    e. In which communities do you deliver services?
    f. How is this organization funded?

            i. Ministry of Health (MOH), or Ministry of Children, Community and Social Services (MCCSS), or other?
            ii. Is it for-profit, or not-for-profit?

6. How are [patients/clients/persons-supported] referred to this agency for services?
    a. What are the eligibility criteria?
    b. How are [patients/clients/persons-supported] discharged or released from the program?
7. Once a [patient/client/person supported] is referred to this organization, what is the general process used to determine the services they receive?
    a. What assessment tools or processes are used?
        i. How are individual goals set?
    b. Are individual needs and preferences incorporated into the plan? And if so how?
        i. Probe: does this process continue to reflect changing needs?
        ii. Who is included in the consultation process?
            1. Family, funders, health care providers, social service providers.
        iii. How long does this process take?
        iv. Who provides final approval?
        v. Do [patients/clients/persons-supported] receive services from multiple health care providers/agencies? How are needs/services coordinated?
        vi. What happens to their care if they are hospitalized?
8. Could you share some benefits of the approach used to determine care plans?
9. Could you share some challenges of the approach used to determine care plans?

10. Can you describe for me an example of a plan that you felt did a good job of addressing the needs of an individual [patient/client/person-supported]?
    a. What makes it a good plan?
    b. How were the [patient/client/person-supported]'s needs and preferences incorporated into the plan?
    c. How was that plan updated to take changes in needs or preferences into account?
11. Could you describe for me an example of a plan that you felt did not do a good job of addressing the needs of an individual [patient/client/person-supported]?
    a. What were the shortcomings of the plan?
    b. How were the [patient/client/person-supported]'s needs and preferences incorporated into the plan?
    c. How is that plan updated to take changes in needs or preferences into account?

Supplementary Material 1: Interview Guide

12. What do you feel are the supports that help your organization develop and deliver care plans?
    a. Funding, staffing
13. What do you feel are some of the barriers that hinder your organization when developing and delivering care plans?
    a. Funding, staffing
14. A [patient/client/person-supported]'s needs can sometimes exceed the funding available to them. How does your organization raise additional funds to support [patients/clients/persons-supported]?
    a. How are these funds raised and distributed?
    b. What are the different sources of funds?
        i. Donations, sponsors, and other government grants
    c. How much of an individual's programming comes from these additional, non-government sources?
    d. What are any additional ways that a [patient/client/person-supported] could increase the funding available for their care?
        i. Jobs, family funding
    e. How do these additional funds affect the funding provided by government sources (e.g. Passport program)?
        i. Clawbacks?
15. How do you pay for extra staff or staffing funding shortfalls?
16. Thinking more broadly about the community-care sector, what do you think are some of the key facilitators and challenges for the organization and delivery of care for your clientele?
    a. For the individual [client/patient/person-supported]?
    b. For organizations?
17. How has/did COVID-19 impact the way you deliver services or create care plans?
    a. Staffing challenges, staff education, pandemic impacts
18. Is there anything else you would like to add?
